# The Who and When of Worry: The Fading Impact of Gender on Worry and Anxiety Severity Among Older Adults

**DOI:** 10.1093/geroni/igaa057.1199

**Published:** 2020-12-16

**Authors:** Katie Granier, Rebecca Ingram, Daniel Segal

**Affiliations:** 1 University of Colorado Colorado Springs, Colorado Springs, Colorado, United States; 2 UCCS, Colorado Springs, Colorado, United States; 3 University of Colorado at Colorado Springs, Colorado Springs, Colorado, United States

## Abstract

Introduction. This study examined the influence of age and gender on diverse worry constructs and overall anxiety among younger (age 18-30) and older (age 65+) adults. Methods. 411 participants (311 younger, 100 older adults; 77.1% female) completed the PSWQ, BMWS, WDQ, and GAS online. Results. Among a series of 2x2 between-subjects ANOVAs, significant interaction effects between age and gender were found among all worry and anxiety measures. Specifically, there was a significant interaction effect on worry severity as measured by the PSWQ (F[1, 393]=4.28, p<.05), the WDQ (F[1, 397]=8.42, p<.01) and the BMWS (F[1, 396]=10.41, p<.01). Gender had a larger impact on worry among younger adults than older, though both age groups showed similar patterns of women reporting greater worry than men. Though both younger and older adults showed a gender difference in worry severity, this difference was mitigated by late life. There was also an interaction effect on anxiety (GAS total) in that younger women reported greater anxiety than younger men but older adults reported similar anxiety across genders, F(1, 384)=9.78, p<.01. Simple main effects analysis showed that younger women scored higher than older women on all measures of worry and anxiety, whereas younger men scored higher than older men on the PSWQ and WDQ but not the BMWS or GAS. Discussion. Consistent with previous literature, women reported greater worry and anxiety than men. However, this difference was mitigated and even extinguished among some measures in older adults. Possible explanations are discussed.

